# What is the current state of clinical evidence for static lung preservation devices, and is it sufficient to inform clinical practice?

**DOI:** 10.1016/j.clinsp.2026.100909

**Published:** 2026-04-11

**Authors:** Anna Luíza Soares de Oliveira Rodrigues, Flavio Pola dos Reis, Thamiris Dias Delfino Cabral, Samuel Lucas dos Santos, Lucas Matos Fernandes, Luis Gustavo Abdalla, Paulo Manuel Pêgo-Fernandes

**Affiliations:** a Department of Surgery, Hospital Universitário Lauro Wanderley, Universidade Federal do Paraíba (UFPB), João Pessoa, PB, Brazil; b Instituto do Coração, Hospital das Clínicas HCFMUSP, Faculdade de Medicina, Universidade de São Paulo, São Paulo, SP, Brazil; c Hospital Federal de Bonsucesso, Rio de Janeiro, RJ, Brazil

**Keywords:** Lung transplantation, Organ preservation, Preservation devices

## Abstract

**Introduction:**

Organ preservation at low temperatures has been described since Alexis Carrel's time in 1912. Since then, organs have been stored in a mixture of frozen saline solution and liquid, with a temperature of around 4 °C. Studies suggest higher temperatures may preserve mitochondrial function, allowing for an extended ischemia time. Various systems are commercially available, each with different preservation principles. This study aims to analyze the underlying parameters of each device.

**Materials and methods:**

A systematic review was conducted using the following databases: PubMed, Embase, and Cochrane Library, searching for clinical trials and observational studies evaluating the use of thermal boxes for lung preservation devices in transplantation. The assessed outcomes were Primary Graft Dysfunction (PGD), ventilation time, length of hospital stay, rejection rate, and survival rate.

**Results:**

The search yielded six studies selected based on title and abstract, including 519 lung transplant cases. The preservation systems evaluated were LUNGguard and MYTEMPF. Our studies assessed LUNGguard, with a total of 298 patients. This system maintained an average temperature of 6 °C (4° to 8 °C), and 51 patients developed grade 3 PGD within 72 h. The mean ventilation time was 25.2 h (25 to 25.5 h), and ICU stay ranged from 8 to 9 days. Rejection was observed in 3 out of 18 patients, and hospital survival was reported in 221 out of 231 patients. MYTEMP 65 HC was evaluated in one study with 70 patients, of whom 4 developed grade 3 PGD within 72 h. The mean ventilation time was 49 h, and the length of hospital stay was 25 days. Kaplan-Meier estimated survival was 94 % (HR = 0.65; 95 % CI 0.26 to 1.6).

**Discussion/conclusion:**

Preservation systems show different performances in lung preservation devices for transplantation. Across available studies, LUNGguard has been reported to maintain temperature stability with corresponding survival findings. Across studies of MYTEMP, lower grade 3 PGD rates have been reported, together with longer ventilation and hospitalization durations, and the device is described as non-transport. Other devices exist on the market, but their data remains unpublished. In summary, ongoing clinical studies with these and other preservation devices are expected to provide new evidence in the near future. As additional data become available, an updated systematic review will help clarify their comparative performance and guide clinical practice.

## Highlights

• Data on LUNGguard indicate stable hypothermic conditions; reported short-term survival is high but based on limited studies.
• A single study on MYTEMP 65 HC reported a lower PGD3 incidence but longer ventilator times.
• Significant evidence gaps exist for most commercial devices, underscoring the critical need for comparative studies.
• Weak evidence suggests varied clinical outcomes across devices.

## Introduction

Lung transplantation success is highly dependent on optimal lung preservation.^1^ For decades, Static Ice Storage (SIS) has been the standard method, favored for its simplicity and low cost.^2^^,^^3^ This technique involves flushing the lungs with a cold preservation solution, inflating them with oxygen, and storing them on ice.^2^ However, the lung tissue is particularly susceptible to temperature-related injury, which can increase the risk of Primary Graft Dysfunction (PGD). Additionally, SIS limits the preservation time to approximately 8 h, thereby restricting its utility in extended procurement scenarios.^4^

PGD remains a leading cause of early mortality following lung transplantation, with severe allograft dysfunction occurring in approximately 30 % of cases.^5^ Although the maximum acceptable preservation time remains undefined, ischemic durations exceeding 8 h are considered high risk and have been associated with poorer post-transplant outcomes.^6^^,^^7^ Known complications include the need for postoperative Extracorporeal Membrane Oxygenation (ECMO) support and prolonged mechanical ventilation.^6^ Recently, growing concerns have emerged regarding the potential impact of organ temperature fluctuations during static ice storage.

Recent studies have demonstrated that storing donor lungs at 10 °C can safely extend preservation times up to 24 h without compromising early postoperative outcomes.^4^^,^^7^^,^^8^ This intermediate temperature appears to preserve mitochondrial health, minimize tissue injury, and improve pulmonary function compared to traditional cold storage at 0 °C.^9^, ^10^, ^11^ In light of these findings, the authors conducted a systematic review to evaluate the use of temperature-controlled thermal boxes for lung preservation devices in the context of transplantation.

## Material and methods

### Protocol and registration

**Fig. 1:**
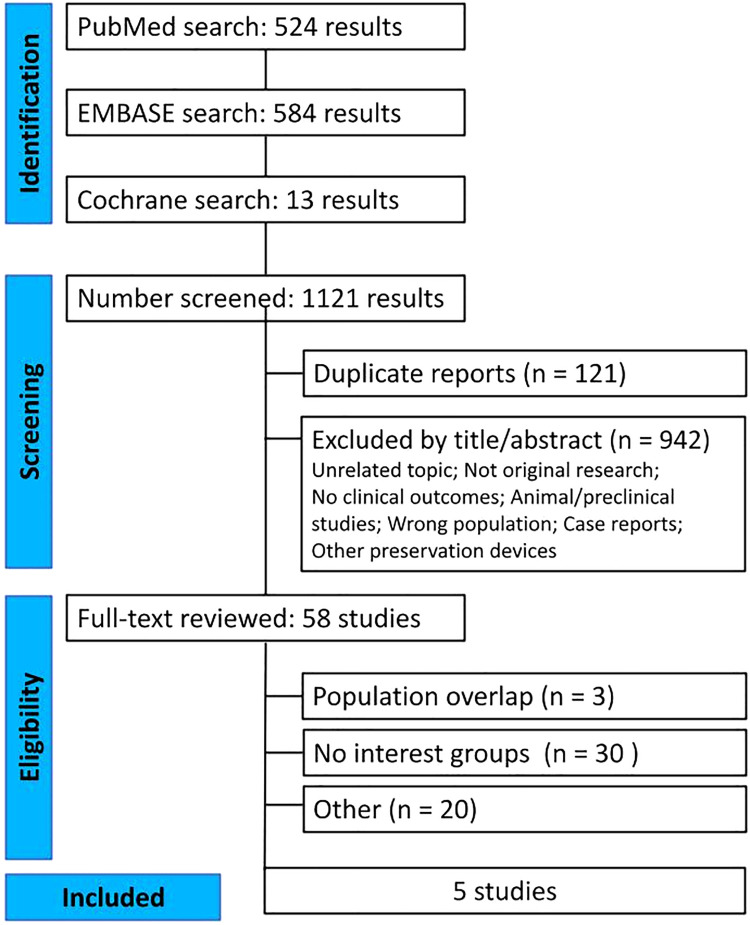
PRISMA flow diagram of study screening and selection. Blue vertical boxes indicate each stage of the screening, and the horizontal boxes present more detailed information about the process, including the steps performed in each stage.

This systematic review and meta-analysis followed the Preferred Reporting Items for Systematic Reviews and Meta-Analysis (PRISMA) guidelines (Fig. 1).^12^ The protocol was prospectively registered in the International Prospective Register of Systematic Reviews (PROSPERO) under the registration number CRD420251083635. The PRISMA checklist for the abstract and the manuscript is available in Supplementary Table S1.

### Eligibility criteria

Studies that met the following eligibility criteria were included: 1) Prospective and retrospective studies (controlled or not); 2) Involved lung transplant recipients; 3) Evaluated thermal preservation systems used during graft preservation. The authors excluded: 1) Overlapping populations; 2) Without outcomes of interest (e.g., primary graft dysfunction, ventilation time, hospital stay, or survival); and 3) With unpublished results or insufficient data for extraction.

To be eligible for inclusion, studies were required to report at least one survival or response outcome as listed above. No specific restrictions were made as to the publication date, presence of a control/comparator group, number of patients, or the treatment regimen assessed.

### Search strategy and data extraction

Pubmed, Cochrane Library, and Embase, were systematically searched on August 20th, 2025. The search strategy included the terms “lung”, “transplantation”, “preservation”, “transplant”, “Controlled Hypothermic Static”, “Portable Preservation”, “hypothermic cold storage”, “Paragonix”, “LUNGguard”, “myTEMP 65HC Incubator”, “E3 CORTEX”, “X Port Lung”.

In addition to the electronic database search, a complementary search was performed using the backward snowballing technique, by manually screening the reference lists of the included studies. However, no additional studies were identified through this method.

Those found in the databases and the references of the articles were incorporated into the reference management software (EndNote®, version X7, Thomson Reuters, Philadelphia, USA). Duplicate articles were removed using both automated and manual methods. Subsequently, two reviewers (A.L.S.O.R. and T.D.D.C.) independently analyzed the titles and abstracts of the identified articles. Disagreements were resolved by consensus between the two authors and senior authors (A.L.S.O.R., T.D.D.C., and F.P.R.).

The following baseline characteristics were extracted: 1) ClinicalTrials.gov Identifier and study design; 2) Number of patients; 3) Incubator model; 4) Donor and recipient’s age; 5) Recipient’s BMI and sex; 6) Lungs transplantated; 7) Donation type; 8) Smoking history; 9) Temperature, 10) Total ischemic time; 11) Donor P/F ratio; 12) Indication for transplant; 13) PGD3 72 h; 14) Time on ventilator; 15) ECMO post-transplant; 16) Length of hospital stay; 17) Follow-up duration. Two authors (A.L.S.O.R. and T.D.D.C.) collected pre-specified baseline characteristics and outcome data.

### Endpoints

Outcomes of interest were: 1) Primary Graft Dysfunction (PGD); 2) Time on the ventilator; 3) Lengths of Stay (LOS); 4) Kaplan-Meier Survival; 5) Use of extracorporeal membrane oxygenation after lung transplantation (Post-LTx ECMO). All studies included in these analyses met the criteria, aiming to provide robust and reliable data on outcomes.

### Risk of bias assessment

The quality assessment of each study was carried out using the Cochrane Collaboration tool for assessing the risk of bias in the Risk of Bias in Nonrandomized Studies of Intervention (ROBINS I).^13^^,^^14^ For each randomized trial, a risk of bias score was assigned, indicating whether it was at a high, low, or unclear risk across five domains: randomization process, deviations from intended interventions, missing outcomes, measurement of outcomes, and selection of reported results. In this assessment, each study was categorized as critical, serious, moderate, or low risk in the seven domains: confounding, selection, classification, deviations from intended interventions, missing data, and selection of reported results. Two authors (A.L.S.O.R. and T.D.D.C.) independently conducted the assessment, and consensus resolved disagreements. To quantify the publication bias, Begg’s rank correlation was used.^15^^,^^16^

## Results

### Study selection and baseline characteristics

A total of 1121 studies were retrieved in our systematic search. After the removal of duplicates and the screening of articles based on titles and abstracts, 58 studies remained and were fully assessed. Of these, 5 studies were included in our analysis: 1 nonrandomized, 2 cohort, and 2 prospective studies.^8^^,^^17^, ^18^, ^19^, ^20^ A total of 361 patients were included in these studies, of which 201 were males. The age of the donor ranged from 35.7 to 61.5 mean years, while the recipient’s age ranged from 50.4 to 61.6 mean years. The recipient’s BMI ranged from 23.2 to 25.8 kg/m^2^ mean with most indications for transplant being either restrictive lung disease or obstructive lung disease, 120 against 116 patients.

The majority of transplants were bilateral, 249 in contrast with only 7 unilateral. The donation type was either Donation after Brain Death (DBD) or Donation after Circulatory Death (DCD). DBD was present in 82 transplants, while DCD was present in 57 transplants. Only two studies presented data on smoking history,^18^^,^^21^ with a positive history in 17.8 % (21) and 42.8 % of patients (Table 1).^18^

**Table 1:** Design and characteristics of studies included in the meta-analysis.

Studies	Design	Incubator model	N° of Patients	Age donor - yr	Age recipient - yr	BMI recipient	Male sex repicient	Lungs transplanted	Donation Type	Smoking History	Indication for transplant ‒ restrictive lung disease	Indication for transplant ‒ obstructive lung disease	Total Ischemic Time	Donor P/F ratio^18^
Ali, 2023 ‒ NCT04616365	Non-randomized clinical trial	MYTEMP65HC	70	52 ± 15.1 mean	61.6 ± 9.8 mean	24.6 ± 3.7 mean	52 (74.3 %)	65 (92.9 %) bilateral / 5 (7.1 %) unilateral	54 (77.1 %) DBD / 16 (22.9 %) DCD	30 yes / 35 no / 5 unknown	39 (55.7 %)	21 (30 %)	1^st^ lung 11h 13 / 2^nd^ lung 13h 49	430 (400‒490) median (17)
Pontula, 2022	Matched cohort study	LUNGguard	18	61.5 mean	NA	NA	NA	NA	DCD	NA	14 (77.8 %)	NA	6h 30 min median	423 mean
Haney 2024 ‒ NCT04930289	Retrospective cohort	LUNGguard	86	35.7 ± 13.4 mean	57.7 ± 12.6 mean	25.8 ± 4.7 mean	51 (59.3 %)	NA	15 (17.6 %) DCD	NA	NA	NA	7h	NA
Provoost 2024	Prospective study	LUNGguard	36	56.2 ± 15.7 mean	57.4 ± 11.4 mean	NA	21 (58.3 %)	33 bilateral / 2 single	28 DBD / 8 DCD	NA	14 ILD	18 COPD + CF	1^st^ lung 13h 38 / 2^nd^ lung 15h 41	413.5 median
Novysedlak, 2024	Prospective study	LUNGguard	151	42.2 ± 14.4 mean	50.4 ± 13.1 mean	23.2 ± 4.6 mean	77 (51 %)	151 bilateral	NA	27 yes / 16 unknown	53 fibrosis + sarcoidosis	77 COPD + CF	1^st^ lung15 h / 2^nd^ lung 17h	448 median

RCT, Randomized Controlled Trial; DBD, Donation after Brainstem Death; DCD, Donation after Circulatory Death; ILD, Interstitial Lung Disease; COPD, Chronic Obstructive Pulmonary Disease; CF, Cystic Fibrosis; N/A, Not Available; P/F, PaO_2_/FiO_2_ ratio.

### Survival and response outcomes

Data for PGD3 within 72 h, time on mechanical ventilation, post-transplant ECMO use, length of hospital stay, and one-year survival are presented in Table 2.

**Table 2:** Outcomes of interest for each device.

Studies	Incubator Model	Temperature	PGD3 72-hours (n)	Time on Ventilator^b^	ECMO post-transplant	Length of Hospital Stay^b^	Kaplan-Meier survival 1-year
Ali, 2023 ‒ NCT04616365	MYTEMP65HC	10 °C	4 (5.7 %)	53.5 ± 40 h	5 (7.1 %)	28.3 ± 15.1 days	94 %
Pontula, 2022	LUNGguard	4.7 median°C	7 (38.9 %) p = 1.00	NA	4 (22 %)	30 days	NA
Haney 2024 ‒ NCT04930289	LUNGguard	4.9 median°C	7 (8.1 %) (p = 0.058)	50.4 ± 45.6 h	15 (17.4 %)	NA	92.7 %
Provoost 2024	LUNGguard	6.5 median°C	12 (33.3 %)	25.5 h (6–526^a^)	5 (13.9 %)	28 days	NA
Novysedlak, 2024	LUNGguard	7 median°C	4 (30 %)	29 h	4 (31 %)	30 days	NA

a Tracheostomy due to failure from weaning.

b Values are reported as mean ± SD or median [IQR] as specified.

Z Primary Graft Dysfunction (PGD3 at 72 h)

The incidence of PGD3 at 72 h varied according to the preservation device used. In studies using the LUNGguard, PGD3 rates ranged from 8.1 % to 38.9 %, with Pontula et al. reporting the highest incidence (38.9 %), although in this study, the ischemia time was longer. In contrast, the study evaluating the MYTEMP65HC device reported a PGD3 incidence of 5.7 %.

### Time on mechanical ventilation

For the LUNGguard device, the median reported times on mechanical ventilation were 35 h. In the MYTEMP65HC group, the reported time was 53.5 h, higher than the average for LUNGguard.

### Post-transplant ECMO use

The use of ECMO after transplantation with the LUNGguard device varied between 13.9 % and 31 %, with an average of approximately 21 % across the four studies reporting this outcome. In the MYTEMP65HC study, the ECMO rate was notably lower, at 7.1 %.

### Length of hospital stay

The median length of hospital stay for patients preserved with the LUNGguard system ranged from 28 to 30 days, with a consistent average of approximately 29 days. For the MYTEMP65HC, the reported median hospital stay was 25 days, slightly shorter than for LUNGguard.

### One-year survival

One-year survival was reported in only two studies. With the LUNGguard device, the survival rate of 92.7 %, and for the MYTEMP65HC, the survival rate was 94 %.

### Quality assessment

**Fig. 2:**
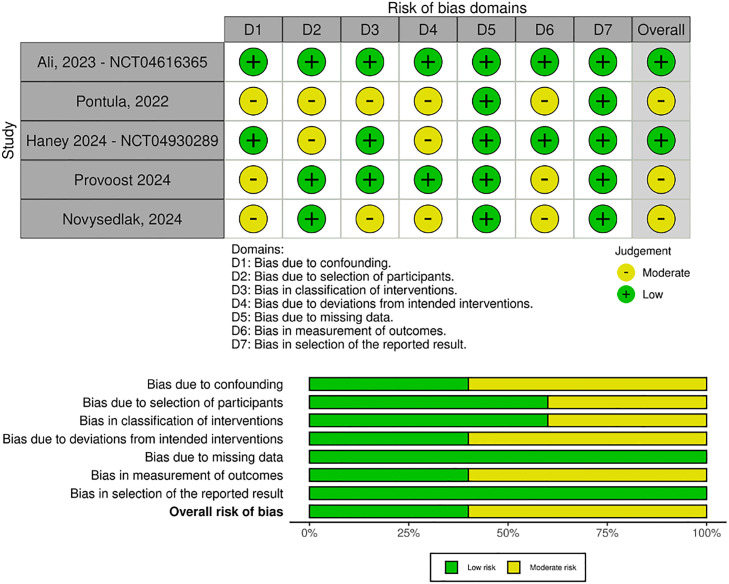
Critical appraisal of studies included according to the Cochrane collaboration’s tool for assessing risk of risk of bias in non-randomised studies ‒ of interventions (ROBINS-I).

The ROBINS-I traffic light diagram (Fig. 2) illustrates the risk of bias for each included nonrandomized study. Studies by Ali 2023 and Haney 2024 were assessed as low risk of bias. Pontula et al. were rated moderate risk due to residual confounding, lack of blinding, and absence of protocol registration, despite robust matching. Measurement of outcomes such as PGD3 and acute rejection also relies on subjective interpretation, adding potential variability.

Provoost et al. and Novysedlak et al. were rated serious risk of bias, primarily due to a lack of control groups or adjustment for confounders. In Provoost et al., participant selection was not matched, and no pre-registered protocol was available. In Novysedlak et al., patients were selected based on ischemic time, introducing selection bias, and no full protocol was reported. Across all studies, patient, donor, and procedural variability contribute to residual confounding, highlighting that the overall quality of evidence is very low.

The ROBINS-I traffic light diagram (Fig. 2) illustrates the risk of individual within-study bias for nonrandomized studies. In the case of studies, such as those conducted by Ali 2023 and Haney 2024, they were determined to be at low risk of bias. However, Pontula et al. was considered to be a moderate risk of bias, while the other two studies had serious concerns due to their biased confounding and participant inclusion selection.

Although Pontula et al. include robust matching, the risk of residual confounding, lack of blinding, and absence of protocol registration prevent it from being rated lower than moderate. Also, measurement of PGD3 and acute rejection relies on subjective components with interpretive variability.

The main limiting factor for Provoost et al. and Novysedlak et al. is the lack of a control group or adjustment. Patient, donor, and procedural variability introduce potential for substantial confounding, being rated as a serious risk of bias in the first domain. While the first has participant selection concerns due to a lack of a matched cohort, the latter included patients based on the ischemic time, which is outcome-related and introduces selection bias. There is no report of a pre-registered protocol for Provoost et al., and the second study doesn't have a full protocol available.

## Discussion

To our knowledge, this is the first systematic review evaluating clinical outcomes associated with different lung preservation devices used during organ preservation for transplantation. Despite advances in preservation technologies, there is still no consensus on which device best ensures optimal graft viability and improves post-transplant outcomes.

Traditionally, SIS has been the standard for organ preservation.^22^ However, in 2020, the International Society for Heart and Lung Transplantation (ISHLT) advised avoiding direct contact of organs with ice to limit cold-induced cellular injury.^23^ The hypothesis that Controlled Moderate Hypothermia (CHS) results in better post-reperfusion outcomes compared to SIS was initially suggested by Joel Cooper's group in 1989. These studies identified an optimal temperature range around 8–10 °C, potentially allowing safe preservation for up to 24 h.^24^, ^25^, ^26^ Decades later, this hypothesis was revisited by Ali et al. and Cypel et al., who in 2021 published the first clinical report of lung transplants using CHS at 10 °C.^4^ Considering that the ideal preservation temperature should be between 4–10 °C, it is worth noting that with SIS, temperatures may drop below this range, approaching 0 °C.^1^

In this context, moderate hypothermia strategies, maintaining preservation temperatures around 8–10 °C, have been proposed as a potential alternative for tissue preservation. In this review, two devices were identified and qualitatively analyzed: MYTEMP65HC and LUNGguard. The first provides preservation at approximately 10 °C, while the LUNGguard, present in four studies, operated at lower preservation temperatures (4.7–7 °C). Previous research conducted by Abdelnour-Berchtold and Ali et al. with rabbit, canine, and porcine lungs observed better mitochondrial health protection after prolonged storage at 10 °C.^22^^,^^23^

The X°Port Lung Transport, developed by the TRAFEROX industry, is a 10 °C preservation system that is still in the investigation phase.^1^^,^^19^ The device does not have FDA approval and is currently being evaluated in a multicenter, randomized, controlled, non-inferiority trial (NCT05898776), which aims to compare lung preservation at 10 °C using the X°Port device versus standard cold storage.^27^ The study plans to enroll 300 participants, and results are expected to be released later in 2025.^28^ While no clinical data have yet been published to support or evaluate its efficacy and safety in isolation, this trial may have an important impact on future clinical guidelines and practice.

Similarly, the VITALPACK® EVO (E3 CORTEX), a device used for the packaging, transport, and preservation of organs at 2–8 °C, also lacks FDA approval. Although it has been in standard use for organ preservation in France and more recently in Switzerland, there are no published clinical data specifically related to lung preservation. An additional tracking device, VITALTRACK, allows monitoring of temperature and location during transport. Given the lack of regulatory approval in the U.S. and the absence of lung-specific clinical evidence, this system was not included in the primary analysis of our review.

Post-graft dysfunction classified as PGD3 is associated with poorer outcomes after transplantation.^28^^,^^29^ Its significance lies in its ability to detect early functional impairment in the graft, allowing it to distinguish between organ preservation strategies that better mitigate ischemia-reperfusion injury and those associated with higher injury burden.^30^ Among the devices included in this review, MYTEMP65HC reported the lowest PGD3 rate at 72 h (5.7 %), while LUNGguard results varied considerably, ranging from 8.1 % to 38.9 %. However, MYTEMP65HC data were derived from a world-leading transplant center, suggesting that outcomes may reflect institutional expertise, patient selection, and perioperative management rather than intrinsic device performance. Similarly, the higher PGD3 rate reported by Pontula et al. with LUNGguard coincided with longer ischemic times, a well-established risk factor for primary graft dysfunction. Overall, the observed variability across studies likely reflects heterogeneity in study design, donor and recipient characteristics, ischemic times, and differences in the application or timing of PGD3 assessment, rather than true differences in device efficacy. Most available studies are small, retrospective, and methodologically heterogeneous, which limits comparability and underscores the need for standardized definitions and prospective, adequately powered trials.

Other recipient-centered outcomes, such as duration of mechanical ventilation, need for ECMO after surgery, length of hospital stay, as well as one-year survival, are also important indicators of transplant success and overall patient recovery.^28^

The typical duration of mechanical ventilation after lung transplantation varies depending on patient factors, perioperative course, and presence of complications, such as PGD and need for extracorporeal support. While the median duration of mechanical ventilation is 41 to 59 h, another study has reported that most patients are extubated within the first few days postoperatively. With the strong association between prolonged intubation and increased mortality, there is an effort to reduce VM duration. When analyzing the results for both devices, they had similar findings, with time on ventilator ranging from 25.5 to 53.5 h.^29^

Furthermore, the ECMO post-transplantation plays a crucial role in rescue therapy, primarily for managing PGD and refractory hypoxemia. It provides effective oxygenation and ventilation while maintaining allograft perfusion.^23^ Evidence supports that ECMO should be started early on, particularly in patients with risk factors, including pre-existing pulmonary hypertension, elevated pulmonary artery pressures, and intraoperative instability.^8^ Although it can reduce the incidence of irreversible graft failure and multiorgan dysfunction, patients requiring ECMO continue to experience lower long-term survival rates compared to those who do not.^23^ Among the included devices, MYTEMP65HC had the lowest need for post-transplant ECMO (7.1 %), while LUNGguard ECMO rates ranged between 13.9 % and 31 %. Our findings show that the LUNGguard device presents a higher need for ECMO; however, the other device was used only by one study, which limited this comparison.

The one-year survival rate is the primary metric for assessing transplant center performance in the US and internationally, shaping both inter-center comparisons and Q patient counseling.^30^, ^31^, ^32^ Clinically, survival beyond the first year is strongly associated with improved long-term outcomes.^30^^,^^32^ In our analysis, only a few studies reported one-year survival data with a favorable outcome. Both devices demonstrated one-year survival rates exceeding 90 %, with MYTEMP65HC at 94 % and the single LUNGguard study reporting 92.7 %, suggesting comparable short-term survival performance.

Moreover, the length of hospital stay is also variable in the literature, with a median of 16 to 30 days, and a significant proportion of patients experience prolonged stays beyond 25 days.^33^ This may be seen especially in high-risk and lobar lung transplant populations, ranging from 28.5 to 58 days.^34^ Prolonged LOS, just like the previous outcomes, is associated with increased early and late mortality after lung transplantation.^33^ Although both devices had a similar LOS, with the longer duration being 30 days, MYTEMP65HC presented the smallest median, with only 25 days.

In general, the available data suggest that higher preservation temperatures, close to 10 °C, may be associated with better outcomes, such as a lower incidence of PGD3 and a reduced need for ECMO. However, it is still unclear whether these advantages depend exclusively on the device used or whether they are a direct consequence of strict temperature control or confounding factors such as the surgeon’s experience. Given this, the question remains: does any device, regardless of the model, offer a real benefit over conventional preservation on ice? The answer to this question depends on direct comparative studies, which are still scarce in the literature.

This systematic review has limitations. First, the number of available studies remains small, and considerable variability exists among them regarding preservation protocols and study designs. These differences limited direct comparisons between devices and precluded the possibility of conducting a meta-analysis. Secondly, no randomized controlled trials directly comparing preservation devices are currently available, representing a gap in the literature. Thirdly, although high survival rates were observed, this finding is based on only two studies, limiting the ability to draw robust or comparative conclusions. Future studies with larger and more diverse cohorts are needed to validate these preliminary results and clarify the true impact on survival outcomes. Fourthly, many confounding factors involved in the transplant process were not assessed due to a lack of information. Among the main confounding factors are institutional protocols and surgeon experience that may influence the surgery outcome more than the device itself. It is important to note that no statistical synthesis was performed in this review. This decision was due to the small number of included studies and the lack of a common control group across them, which prevented meaningful quantitative comparisons. As a result, sensitivity analysis to explore the impact of potential sources of bias was not applicable. This limitation may have affected the interpretation of the findings and highlights the need for caution when drawing general conclusions from the current evidence.

## Conclusion

Rising concern about optimal preservation temperatures in lung transplantation has led to the development of devices to extend preservation time and improve outcomes. This systematic review summarizes the current clinical evidence on static lung preservation devices, highlighting reported outcomes such as graft function, hospital stay, and survival. While MYTEMP65HC was associated with favorable PGD3 rates in one study, the evidence base is still limited and heterogeneous, making direct comparisons between devices inappropriate. Nevertheless, the findings provide a valuable overview of the existing literature and emphasize areas where further research is needed. Well-designed, prospective studies are essential to better understand the performance and safety of these devices and to guide clinical practice.

## Ethical approval

No ethical approval was required for this systematic review with meta-analysis, as all data were already published in peer-reviewed journals. Furthermore, no patients were involved in the design, conduct, or interpretation of our study.

Correspondence and requests for materials should be addressed to F.P.R; flavio.pola@hc.fm.usp.br.

## Authors' contributions

All authors contributed to the study conception and design. [A.L.S.O.R and F.P.R.] conceived the project, and material preparation, data collection, and analysis were performed by [A.L.S.O.R., T.D.D.C., S.L.S.]. The figures and tables were created by [A.L.S.O.R; T.D.D.C.; L.M.F; L.G.A]. The first draft of the manuscript was written by [A.L.S.O.R, T.D.D.C.; F.P.R; P.M.P.F] and all authors commented on previous versions of the manuscript. All authors read and approved the final manuscript.

## Data availability statement

Data is contained within the article.

## Conflicts of interest

The authors declare no conflicts of interest.

## Appendix Supplementary materials

Image, application 1
